# A three-minute all-out test performed in a remote setting does not provide a valid estimate of the maximum metabolic steady state

**DOI:** 10.1007/s00421-022-05020-3

**Published:** 2022-08-10

**Authors:** Ed Maunder, Jeffrey A. Rothschild, Andrius Ramonas, Matthieu Delcourt, Andrew E. Kilding

**Affiliations:** 1 Sports Performance Research Institute New Zealand, Auckland University of Technology, Auckland, New Zealand; 2 Department of Biology, University of Ottawa, Ottawa, Canada

**Keywords:** Cycling, Threshold, Reliability, Validity, Remote

## Abstract

**Purpose:**

The three-minute all-out test (3MT), when performed on a laboratory ergometer in a linear mode, can be used to estimate the heavy–severe-intensity transition, or maximum metabolic steady state (MMSS), using the end-test power output. As the 3MT only requires accurate measurement of power output and time, it is possible the 3MT could be used in remote settings using personal equipment without supervision for quantification of MMSS.

**Methods:**

The aim of the present investigation was to determine the reliability and validity of remotely performed 3MTs (3MT_R_) for estimation of MMSS. Accordingly, 53 trained cyclists and triathletes were recruited to perform one familiarisation and two experimental 3MT_R_ trials to determine its reliability. A sub-group (*N* = 10) was recruited to perform three-to-five 30 min laboratory-based constant-work rate trials following completion of one familiarisation and two experimental 3MT_R_ trials. Expired gases were collected throughout constant-work rate trials and blood lactate concentration was measured at 10 and 30 min to determine the highest power output at which steady-state ${{\dot{V}O}}_{2}$ (MMSS-${{\dot{V}O}}_{2}$) and blood lactate (MMSS-[La^−^]) were achieved.

**Results:**

The 3MT_R_ end-test power (EP_remote_) was reliable (coefficient of variation, 4.5% [95% confidence limits, 3.7, 5.5%]), but overestimated MMSS (EP_remote_, 283 ± 51 W; MMSS-${{\dot{V}O}}_{2}$, 241 ± 46 W, *P* = 0.0003; MMSS-[La^−^], 237 ± 47 W, *P* = 0.0003). This may have been due to failure to deplete the finite work capacity above MMSS during the 3MT_R_.

**Conclusion:**

These results suggest that the 3MT_R_ should not be used to estimate MMSS in endurance-trained cyclists.

## Introduction

The maximal metabolic steady state (MMSS) demarcates the transition from heavy to severe-intensity exercise (Jones et al. 2019). During severe-intensity exercise, blood lactate concentration, whole-body oxygen consumption (${{\dot{V}O}}_{2}$), muscle [H^+^], and muscle [PCr] cannot stabilise, and task failure is characterised by consistent perturbations in these values and attainment of peak ${{\dot{V}O}}_{2}$ (${{\dot{V}O}}_{2}$peak). In the heavy domain, these muscle and whole-body parameters attain a delayed steady state (Black et al. 2017). The MMSS has been assessed using the critical power model (Jones et al. 2019). Using ~ 3–5 time-to-task failure severe-intensity trials lasting ~ 2–15 min, critical power is identified as the power-asymptote of the hyperbolic power–duration relationship, and the finite work capacity above critical power as the curvature constant (W′) (Jones and Vanhatalo 2017; Jones et al. 2019). Critical power measured in this manner has been shown to discriminate heavy and severe-intensity exercise responses, and therefore estimate the MMSS (Jones et al. 2008; Black et al. 2017). Work output at the MMSS is used in training intensity regulation, training load monitoring, and predicting endurance performance (Coyle et al. 1988; Maunder et al. 2021).

The requirement for multiple time-to-task failure severe-intensity trials led to development of the three-minute all-out test (3MT) for identification of the MMSS (Burnley et al. 2006; Vanhatalo et al. 2007). In the 3MT, an athlete works all-out for three minutes without pacing, depleting W′ in the initial part of the test to ensure the work output is eventually limited to the critical power. Accordingly, average power output during the final 30 s is used to estimate critical power, and the total work performed above the end-test power is used to estimate W′. The 3MT was designed for use on an electromagnetically braked laboratory ergometer in a linear mode, where power output is the product of the linear factor (flywheel resistance) and the square of the cadence; the linear factor is typically applied, such that the power output achieved at the individual cyclist’s preferred cadence is the gas exchange threshold power output (GET) plus 50% of the interval between GET and ${{\dot{V}O}}_{2}$peak (Burnley et al. 2006; Vanhatalo et al. 2007, 2008a). The athlete is also supervised and verbally encouraged by a team of researchers, and blinded to power output and time (Burnley et al. 2006; Vanhatalo et al. 2007, 2008a). The 3MT performed in this manner has been shown to produce valid (Burnley et al. 2006; Vanhatalo et al. 2007, 2016) and reliable (Burnley et al. 2006; Johnson et al. 2011) estimates of MMSS and W′ in trained populations.

Since development of the laboratory-based 3MT in 2006, the availability of accurate and reliable power-measuring devices for use during indoor cycle training has increased dramatically (Hoon et al. 2016; Zadow et al. 2016, 2018). As the 3MT only requires accurate measurement of power output and time, it is theoretically possible that the 3MT could be used by athletes in remote settings using personal equipment without supervision for quantification of MMSS and W′. However, it is unknown if the 3MT provides reliable and valid estimates of the MMSS when performed by unsupervised endurance athletes remotely, using typical indoor training set-ups, where there is opportunity to shift gears and therefore resistance to pedalling, and view elapsed time (3MT_R_). Addressing this gap in the literature is pertinent for remote endurance coaches operating primarily without face-to-face communication with athletes, and for endurance athletes without regular access to laboratory facilities.

Therefore, the primary aim of the present investigation was to determine the reliability and validity of the 3MT for estimation of the MMSS when performed in remote settings by unsupervised athletes using their own indoor cycling setup. It was hypothesised that the 3MT_R_ would produce reliable and valid estimates of the MMSS.

## Materials and methods

### Participants

Fifty-three trained cyclists and triathletes completed the present investigation (32 males, 21 females; age, 39 ± 9 y; self-reported training volume, 10 ± 3 h^.^week^−1^). Prospective participants were recruited via social media, and any healthy cyclist or triathlete training > 5 h^.^week^−1^ with access to power-measuring devices (e.g., smart indoor trainer, power pedals, and power cranks) was eligible to take part in the study. After reading a participant information sheet hosted online (Qualtrics, Provo, UT, USA), participants provided informed consent and were directed to a health screening, and, if passed, to a survey to provide details on their anthropometry, basic training history, and training equipment. The survey ended with specific instructions for how to complete the 3MT_R_ trials. Participants were able to complete the trials using their road bicycle mounted to a “rear wheel off” indoor trainer, in which the rear wheel is removed and the bike is attached to the cassette of the static trainer (*N* = 38), or “rear wheel on” indoor trainer, in which the rear wheel is in contact with a roller (*N* = 15). The 3MT_R_ trials were completed by participants in their home training set-ups without the researchers present. All procedures were approved by the Auckland University of Technology Ethics Committee (20/137).

### Remote trials

Participants performed the remote 3MT_R_ on three occasions: one familiarisation trial and two experimental trials, each separated by 4–10 days. In advance of the two experimental trials, participants were asked to refrain from vigorous exercise for 24 h and caffeine ingestion for 1 h and repeat any caffeine ingestion within 12 h of the first trial. Participants were asked to complete all trials using the equipment they detailed in the survey and wear a heart rate monitor throughout. These pre-trial controls were designed to simulate what a coach in remote settings could realistically achieve.

Participants were asked to warm up for 10 min at 100 W before commencing each 3MT_R_. The 3MT_R_ was a three-minute all-out effort, in which the participant was asked to produce their maximum power output at every moment of the test. Participants were able to shift gears during the trials. The participant information sheet described the expected pattern of power output vs. time, with power output first rising to a peak before steadily declining and levelling off in the second half of the test. Participants were asked to perform a self-selected cool down following each 3MT_R_. Following each trial, participants emailed output files to the researchers. Familiarisation trial files were screened to ensure that the test was completed appropriately. This included ensuring the overall power output vs. time curve matched the expected profile, and any inexplicable rises in power output late in the test that would identify pacing. In all correspondences, participants were reminded that trials were to be completed in an all-out, unpaced fashion, such that the maximum possible power output was being produced at every moment of the test. Peak, time-to-peak, mean, end-test (EP_remote_; average over the last 30 s), and lowest (average over 6 and 30 s) power output were calculated for each 3MT_R_, along with total work done, work done above EP_remote_ (WEP_remote_), and second-by-second power output and cadence using TrainingPeaks WKO + (Peaksware, LLC, Lafayette, USA).

### Laboratory validity trials

A sub-group (*N* = 10) of locally based participants reported to the laboratory on 4–6 occasions following completion of three 3MT_R_ trials (8 males, 2 females; mass, 71 ± 13 kg; height, 178 ± 10 cm; self-reported training volume 9 ± 3 h^.^week^−1^; ${{\dot{V}O}}_{2}$peak, 54 ± 7 mL^.^kg^−1.^min^−1^). These participants first completed an incremental exercise test for determination of ${{\dot{V}O}}_{2}$ peak. Briefly, participants commenced cycling on an electromagnetically braked ergometer (Excalibur Sport, Lode BV, Groningen, The Netherlands) at 60 W. The work rate increased by 30 W every minute until task failure, with continuous collection of expired gases (TrueOne 2400, ParvoMedics, UT, USA). The ${{\dot{V}O}}_{2}$peak was accepted as the highest 15 s average ${{\dot{V}O}}_{2}$.

Participants returned to the laboratory for three-to-five constant-work rate trials 4–10 days apart, at the same time of day as the 3MT_R_ trials, having refrained from vigorous exercise for 24 h and caffeine for 1 h, and replicated their self-reported 24 h dietary intake, to identify the power output at the MMSS. These trials were completed on each participant’s own equipment, and the same equipment they used to complete the 3MT_R_ trials. These trials were completed on an “ergometer mode” on a smart trainer, such that the work-rate was held constant throughout, and participants were instructed to maintain their preferred cadence. The sub-group of participants completing the validity phase of the study used either a Wahoo Kickr (*N* = 9, Wahoo Fitness, Atlanta, USA) or Tacx Neo 2 T (*N* = 1, Garmin^®^, KS, USA) to measure power output. The reliability and validity of the Wahoo Kickr has been established (Hoon et al. 2016; Zadow et al. 2016, 2018), whereas the Tacx Neo 2 T has to our knowledge not been validated in research; however, the data from this individual participant support credible reliability within this study (within-subject coefficient of variation for ${{\dot{V}O}}_{2}$ during 5–8 min of the standardised warm-up in the validity trials, 2.5%).

Constant-work rate trials began with a 10 min standardised warm-up of 50% of remote end-test power (EP_remote_) for 8 min, followed by 1 min at 60% and 1 min at 70% EP_remote_, after which the main 30 min trial began. Expired gas was continuously measured using a metabolic cart (TrueOne 2400, ParvoMedics, UT). Gas analysis data were initially visually inspected and aberrant points laying more than three standard deviations from the local mean were removed and a three-point moving average filter was applied to the data set. The ${{\dot{V}O}}_{2}$ response kinetics was modelled using exponential and linear fitting to determine the presence (or absence) of a ${{\dot{V}O}}_{2}$ slow component (Eq. 1). The amplitude of slow component was calculated by taking the difference between the steady-state value of the fundamental component and the average ${{\dot{V}O}}_{2}$ in the final 60 s of the constant-work rate trial. Steady-state ${{\dot{V}O}}_{2}$ was defined by a slow component amplitude less than the within-subject coefficient of variation for ${{\dot{V}O}}_{2}$ during minutes 5–8 of the warm-up (3.2%). Additionally, duplicate capillary blood lactate samples were obtained from a finger after 10 and 30 min, with steady-state blood lactate concentrations defined as a rise of < 1 mmol^.^L^−1^ from 10 to 30 min

1 $$\dot{V}{\text{O}}_{2} \left( t \right) \, = \, \dot{V}{\text{O}}_{{2{\text{baseline}}}} + A_{{\text{p}}} \left[ {1 - e^{{ - \left( {t - TDp} \right)/\tau p}} } \right] \, + \, S \, \left[ {t \, - \, TD_{{\text{s}}} } \right],$$

where ${{\dot{V}O}}_{2}$(*t*) is the absolute ${{\dot{V}O}}_{2}$ at a given time *t*, ${{\dot{V}O}}_{2}$ represents the mean ${{\dot{V}O}}_{2}$ during 6-8 min of the warm-up, and A_p_ and τ_p_ represent the amplitude and time constant of the primary component of ${{\dot{V}O}}_{2}$ kinetics, TD_s_ is a time delay of slow component, and S (slope) is a coefficient of linear regression.

The first trial was performed at EP_remote_. Subsequent trials were completed with power outputs ± 2.5% of EP_remote_ until at least one trial with ${{\dot{V}O}}_{2}$ steady-state and non-steady-state characteristics had been performed. The mid-point of the highest power output at which a ${{\dot{V}O}}_{2}$ steady-state was achieved and the lowest power output exhibiting non-steady-state ${{\dot{V}O}}_{2}$ behaviour was accepted as the maximum ${{\dot{V}O}}_{2}$ steady-state (MMSS-V̇O_2_), and the mid-point of the highest power output at which steady-state blood lactate concentrations were observed and the lowest power output exhibiting non-steady-state blood lactate concentrations was accepted as the maximum lactate steady state (MMSS-[La^−^]). The validity of EP_remote_ was assessed in this manner, rather than against the laboratory-based 3MT or critical power derived from a series of severe-intensity constant-work rate trials to task failure, to provide a direct measure of the MMSS.

### Statistical analysis

Data are presented as mean ± standard deviation (SD) unless otherwise stated. Data were assessed for normality using the Shapiro–Wilk test. Simple comparisons were made using paired *t* tests (or non-parametric equivalents). The reliability of EP_remote_, total work done (TWD), and work above end power (WEP_remote_) was assessed using within-SD coefficients of variation (CV) and Pearson’s correlation coefficients, both expressed with 95% confidence intervals. Hedges’ *g* effect sizes (ES) and associated 95% confidence intervals are presented where appropriate. All statistical analyses were carried out with R version 4.0.3 (The R foundation for Statistical Computing, Vienna, Austria). Significance was inferred when *P* ≤ 0.05.

## Results

### *Characteristics of the 3MT*_*R*_

**Fig. 1:**
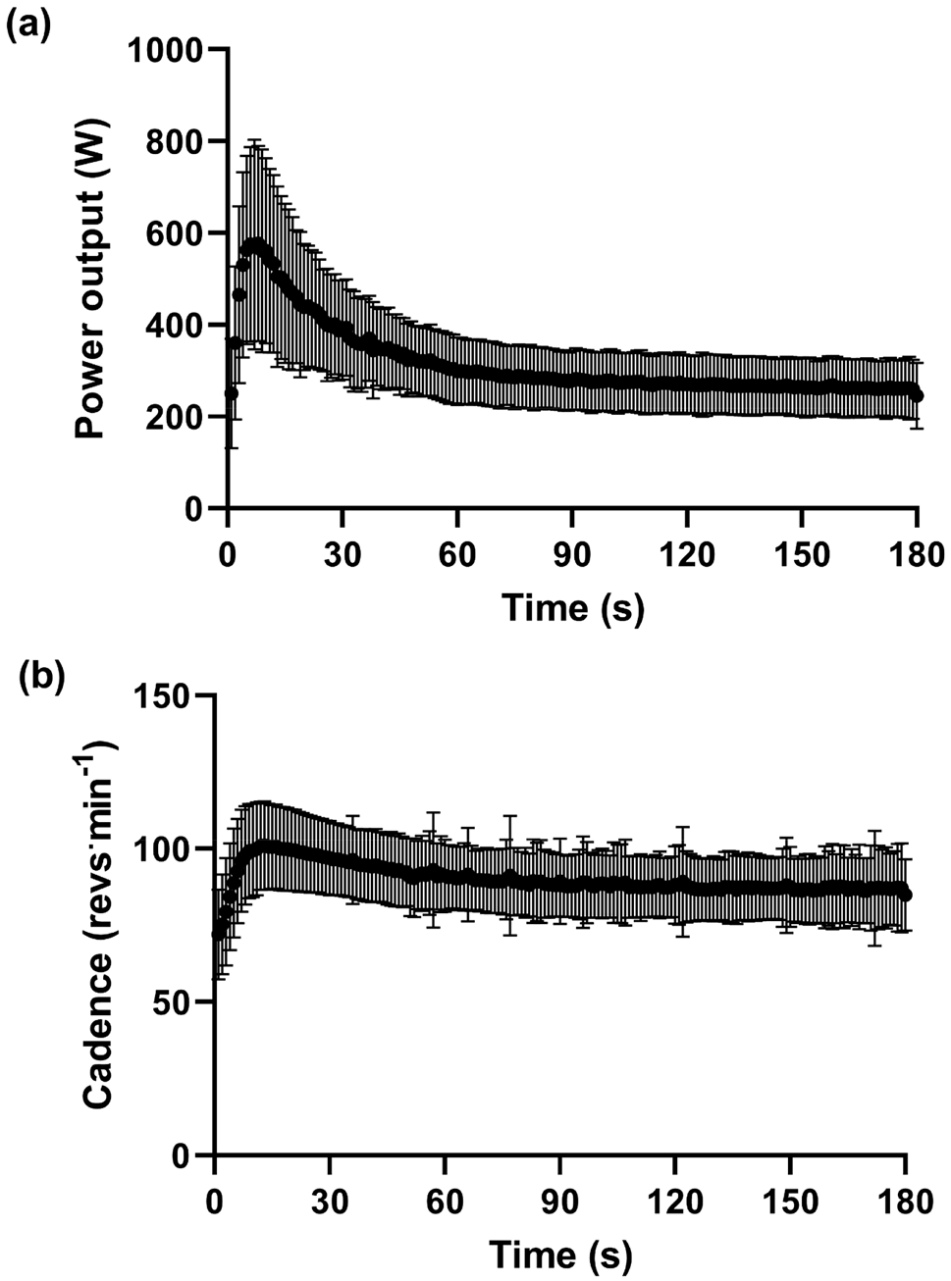
Remote three-minute all-out test **a** mean ± SD power (W) vs. time and **b** mean ± SD cadence (revs^.^min^−1^) vs. time. The mean of each individual’s power and cadence from the two trials was calculated for each second-by-second interval. Data presented are from the whole cohort (*N* = 53)

Power output profiles were consistent between the two 3MT_R_ trials (peak power output, 660 ± 216 vs. 663 ± 238 W; time-to-peak power output, 7 ± 4 vs. 8 ± 6 s; mean power output, 316 ± 73 vs. 318 ± 75 W; EP_remote_, 258 ± 60 vs. 261 ± 63 W; WEP_remote_, 10.4 ± 5.0 vs. 10.1 ± 4.8 kJ; in all cases, *P* > 0.05). Using the mean of each participant’s two trials, 97 ± 9% of W′ was depleted in the first 90 s of the 3MT_R_, the lowest 6 s power output was 91 ± 5% of EP_remote_, and the lowest 30 s power output was 97 ± 3% of EP_remote_. Cadence profiles were consistent between the two 3MT_R_ trials (peak cadence, 110 ± 21 vs. 110 ± 23 revs^.^min^−1^; mean cadence, 90 ± 11 vs. 90 ± 12 revs^.^min^−1^; end 30 s cadence, 86 ± 12 vs. 87 ± 12 revs^.^min^−1^; in all cases, *P* > 0.05). Mean second-by-second power output and cadence profiles of the two 3MT_R_ trials are shown in Fig. 1.

### *Reliability of the 3MT*_*R*_

**Fig. 2:**
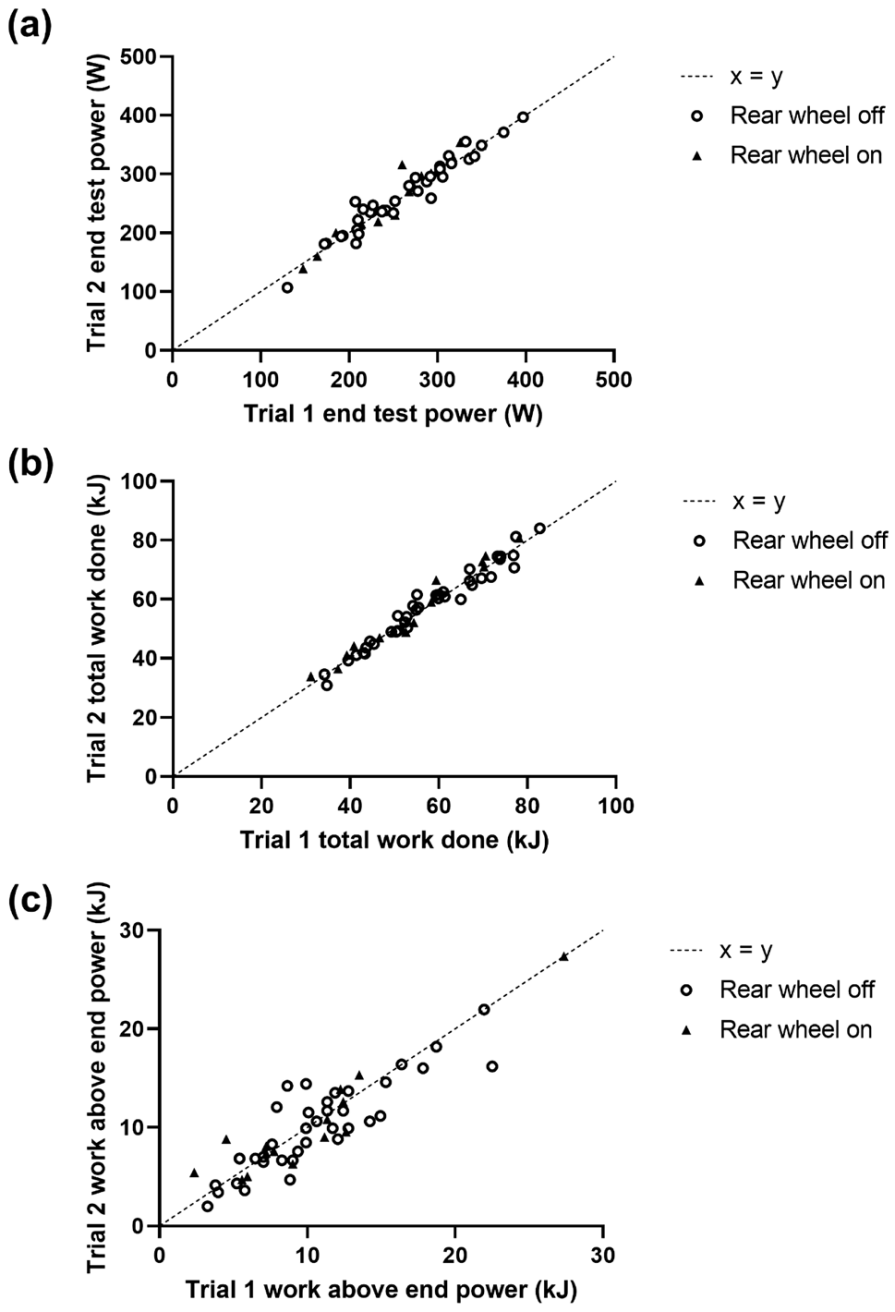
Scatter plot of metrics obtained from the two remote three-minute all-out tests (with dashed line for x = y); **a** EP_remote_ (W), **b** total work done (kJ), and **c** W′ (kJ). Participants completing the trials on a rear wheel off indoor trainer are shown with solid squares. Participants completing the trials on a rear wheel on indoor trainer are shown with x symbols

The EP_remote_ and TWD was acceptably reliable, as evidenced by the lack of systematic variance between the first and second trials, low CV, and strong correlation between-trials; the WEP_remote_ derived from the 3MT_R_ was less reliable (Fig. 2, Table 1). These reliability statistics were largely consistent when considering the overall cohort (*N* = 53), participants who used “rear wheel off” indoor trainers (*N* = 38), and participants who used “rear wheel on” indoor trainers (*N* = 15) (Fig. 2, Table 2). The most common device used to measure power output during the remote 3MT (*N* = 18) was the Wahoo Kickr Core (Wahoo Fitness, Atlanta, USA), which has been validated (Hoon et al. 2016) Reliability statistics performed on this sub-group of participants produced the same inferences as the overall cohort for EP_remote_ (264 ± 64 vs. 264 ± 60 W [*P* = 0.84]; CV, 3.3% [2.4, 3.7%]; *r*, 0.98 [0.94, 0.99, *P* < 0.0001]), TWD (59 ± 14 vs. 58 ± 14 kJ [*P* = 0.60]; CV, 3.2% [2.3, 4.6%]; *r*, 0.98 [0.95, 0.99, *P* < 0.0001]), and W′ (11.0 ± 5.1 vs. 10.8 ± 4.5 kJ [*P* = 0.73]; CV, 17.9% [12.5, 26.8%]; *r*, 0.83 [0.60, 0.94, *P* < 0.0001]).

**Table 1:** Reliability statistics for the remote three-minute all-out test

	Trial 2–Trial 1	CV (95% CI)	*r* (95% CI)
Overall cohort (*N* = 53)			
EP_remote_ (W)	4 ± 16 (*P* = 0.12)	4.5% (3.7, 5.5%)	0.97 (0.94, 0.98, *P* < 0.0001)
TWD (kJ)	0 ± 3 (*P* = 0.38)	3.3% (2.7, 4.0%)	0.98 (0.87, 0.99, *P* < 0.0001)
WEP_remote_ (kJ)	− 0.3 ± 2.3 (*P* = 0.33)	15.6% (12.5, 19.8%)	0.90 (0.82, 0.94, *P* < 0.0001)
Rear wheel off (*N* = 38)			
EP_remote_ (W)	2 ± 15 (*P* = 0.38)	4.0% (3.2, 5.1%)	0.97 (0.94, 0.98, *P* < 0.0001)
TWD (kJ)	0 ± 3 (*P* = 0.84)	3.1% (2.5, 4.0%)	0.98 (0.96, 0.99, *P* < 0.0001)
WEP_remote_ (kJ)	− 0.5 ± 2.3 (*P* = 0.22)	16.2% (12.5, 21.3%)	0.87 (0.77, 0.93, *P* < 0.0001)
Rear wheel on (*N* = 15)			
EP_remote_ (W)	7 ± 19 (*P* = 0.17)	5.5% (3.8, 8.1%)	0.96 (0.89, 0.99, *P* < 0.0001)
TWD (kJ)	1 ± 3 (*P* = 0.07)	3.7% (2.6, 5.5%)	0.98 (0.95, 0.99, *P* < 0.0001)
WEP_remote_ (kJ)	0.1 ± 2.0 (*P* = 0.83)	13.9% (9.1, 23.1%)	0.94 (0.82, 0.98, *P* < 0.0001)

*CI* confidence intervals, *CV* within-subject coefficient of variation, *EP*_*remote*_ average power during the last 30 s of the three-minute all-out test, *r* Pearson’s correlation coefficient, *TWD* total work done, *W′* work above EP_remote_

### *Validity of the 3MT*_*R*_

The sub-group of ten participants who completed the laboratory-based validity component of this study produced similar 3MT_R_ results as the overall cohort (peak power output, 693 ± 255 W; EP_remote_, 283 ± 51 W; WEP_remote_, 9.4 ± 2.6 kJ). None of the participants were able to complete 30 min of constant-work rate cycling at EP_remote_ (time-to-task failure, 11.4 ± 6.5 min; range, 5.0–21.5 min). In all participants, physiological responses to constant-work rate cycling at EP_remote_ were characteristic of the severe-intensity domain (blood [La^−^] at task failure, 12.3 ± 3.5 mmol^.^L^−1^, range, 6.0–18.3 mmol^.^L^−1^; in all cases, the fundamental phase of the ${{\dot{V}O}}_{2}$ response was not completed prior to task failure).

**Fig. 3:**
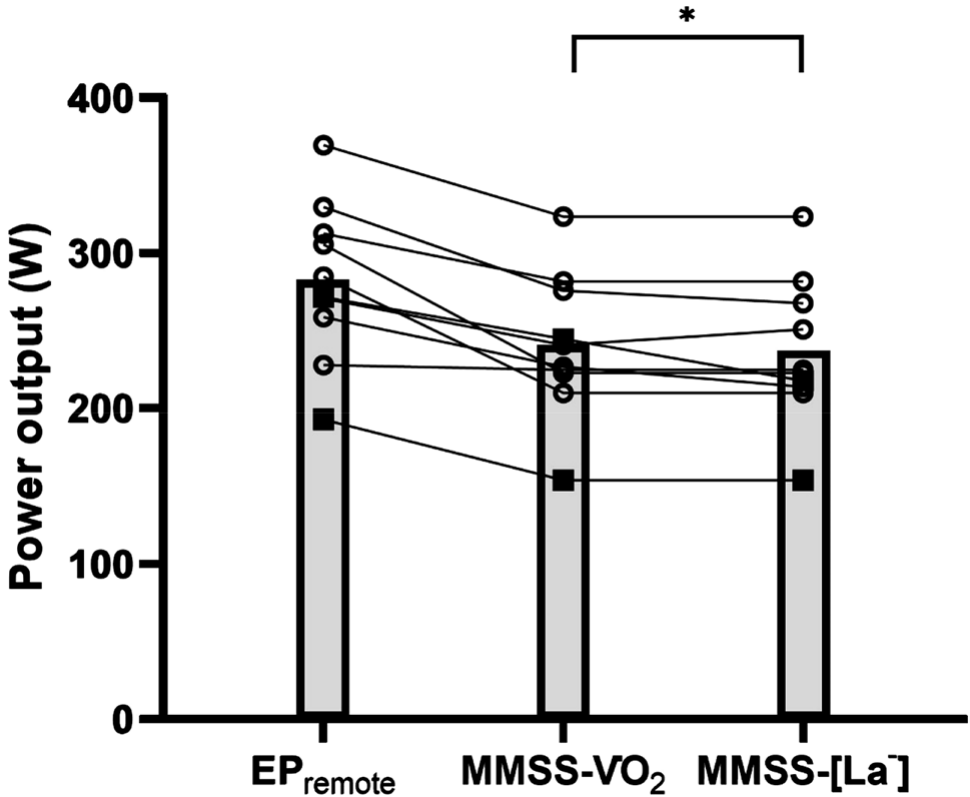
Mean (bars) and individual (lines with markers) values for the end power output during the remote three-minute all-out tests (EP_remote_), power output at the maximum metabolic steady state as defined by ${{\dot{V}O}}_{2}$ kinetics during constant-work rate cycling (MMSS-V̇O_2_), and power output at the maximum metabolic steady state as defined by blood lactate responses to constant-work rate cycling (MMSS-[La^−^]). Male participants are indicated with clear markers (*N* = 8), and female participants are indicated with solid markers (*N* = 2). ‘*’ denotes *P* < 0.05 vs. EP_remote_

**Fig. 4:**
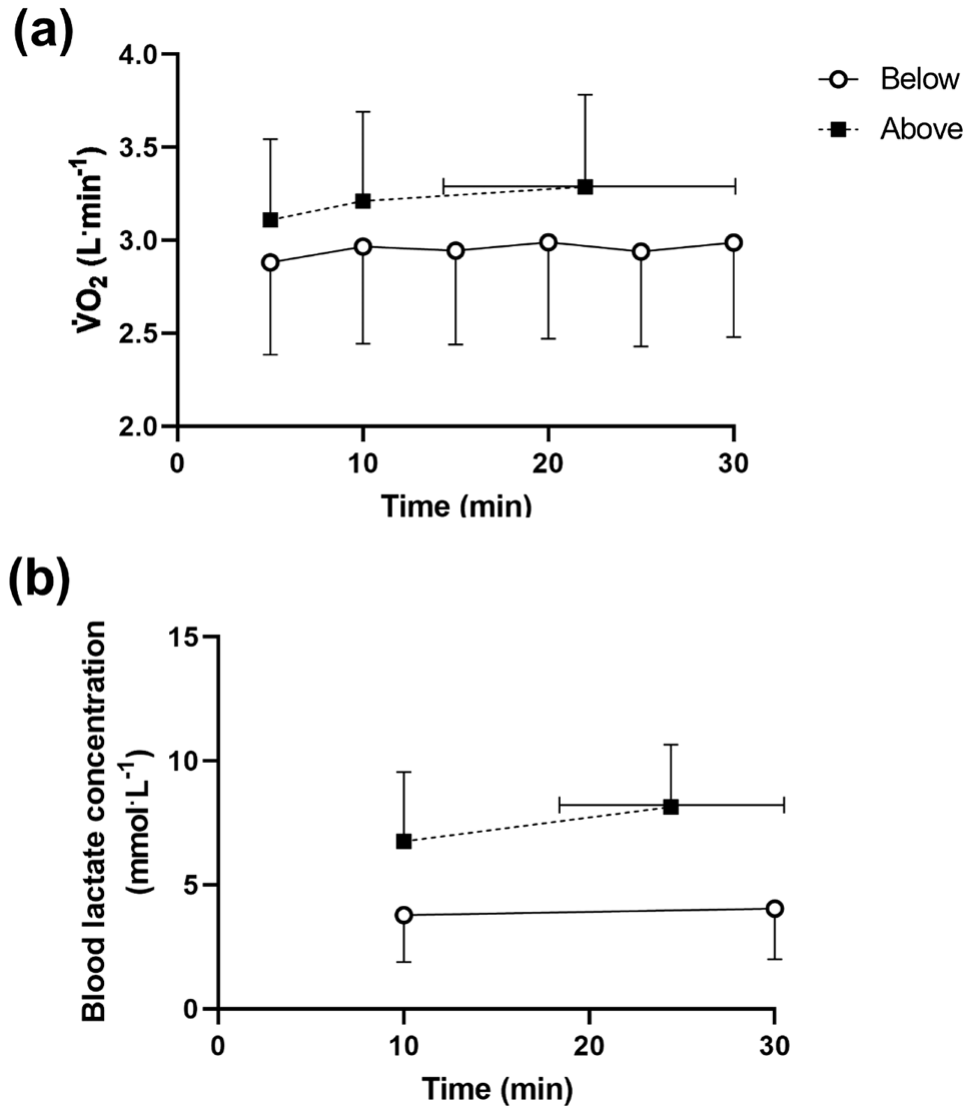
**a** ${{\dot{V}O}}_{2}$ and **b** blood lactate concentration responses to constant-work rate cycling performed immediately below (clear markers) and above (solid markers) **a** MMSS-${{\dot{V}O}}_{2}$ and **b** MMSS-[La^−^] (*N* = 10).

The EP_remote_ overestimated MMSS-${{\dot{V}O}}_{2}$ (241 ± 46 W, *P* = 0.0003, ES = 1.62 [0.71, 2.67], percent difference, 18 ± 11%) and MMSS-[La^−^] (237 ± 47 W, *P* = 0.0003, ES = 1.72 [0.78, 2.82], percent difference, 20 ± 12%). The MMSS-${{\dot{V}O}}_{2}$ and MMSS-[La^−^] were not significantly different (*P* = 0.37, ES = 0.34 [− 0.27, 0.97], Fig. 3). The ${{\dot{V}O}}_{2}$ and blood lactate responses to constant-work rate trials immediately above and below MMSS-${{\dot{V}O}}_{2}$ and MMSS-[La^−^] are shown in Fig. 4.

## Discussion

The primary aim of the present investigation was to determine the reliability and validity of the 3MT when performed in remote settings by unsupervised athletes using their own indoor cycling setup (3MT_R_). The primary outcomes were that end-test power in the 3MT_R_ was reliable, but overestimated the MMSS. These results suggest that the 3MT_R_, as performed in the present investigation, should not be used to estimate the MMSS in endurance-trained cyclists.

The reliability of the 3MT_R_ is evidenced by the low CV and high Pearson’s correlation coefficients for the primary outcome metrics (Fig. 2, Table 1). The CV values reported for EP_remote_ (~ 4.0–5.5%) are similar to what has been reported for laboratory-based measurements of end-test power (~ 3–7%) (Burnley et al. 2006; Johnson et al. 2011). The WEP_remote_ was less reliable than EP_remote_ (CV, ~ 13.9–16.2%), which is in line with reliability data for laboratory-based estimates (CV, ~ 20–28%) (Johnson et al. 2011). Therefore, these data suggest that the 3MT_R_ produces similarly repeatable outcomes as the laboratory-based 3MT. However, despite the strong reliability of the 3MT_R_, we present robust evidence that EP_remote_ overestimated the MMSS; this is shown by the severe-intensity physiological responses and short time-to-task failure (11.4 ± 6.5 min; range, 5.0–21.5 min) during constant-work rate cycling at EP_remote_, and the consistency of differences between EP_remote_ and MMSS-${{\dot{V}O}}_{2}$ and MMSS-[La^−^] at an individual level (Fig. 3). Additionally, we feel the magnitude by which EP_remote_ overestimated the power outputs at MMSS-${{\dot{V}O}}_{2}$ (18 ± 11%) and MMSS-[La^−^] (20 ± 12%) permits this conclusion, even in the context of recent data demonstrating the transition from the heavy to severe-intensity domain is a phased transition (Pethick et al. 2020). Therefore, it is unlikely that the overestimation of MMSS by EP_remote_ is attributable to variability in the results of remote tests.

In the 3MT_R_, total work completed above the end-test power was substantially lower (10.3 ± 4.8 kJ) than has typically been observed during laboratory-based trials (~ 14–17 kJ) (Burnley et al. 2006; Vanhatalo et al. 2007, 2008a, 2008b, 2016). Importantly, the sub-group of participants included in the validity aspect of this study had similarly low WEP_remote_ (9.4 ± 2.6 kJ). In these participants’ work above, the MMSS-${{\dot{V}O}}_{2}$ power output was similar to work above laboratory-based end-test power observed elsewhere (17.4 ± 3.7 kJ) (Burnley et al. 2006; Vanhatalo et al. 2007, 2008a, 2008b, 2016). Accordingly, it is likely participants in the present investigation failed to fully deplete work output above MMSS in the initial 150 s of the test, and therefore, that EP_remote_ was supplemented by work output above MMSS. This would explain why subsequent constant-work rate trials at EP_remote_ produced clear severe-intensity responses, and therefore, why EP_remote_ significantly overestimated MMSS.

The reason why work above MMSS was not fully depleted in the initial 150 s of the 3MT_R_ could be attributable to several factors. First, laboratory-based trials are supervised and strong verbal encouragement is provided throughout the test (Burnley et al. 2006; Vanhatalo et al. 2007, 2008a, 2008b). Therefore, in the 3MT_R_ where participants were unsupervised and strong verbal encouragement was not provided by the researchers, it is possible the absence of social facilitation resulted in a sub-maximal or paced effort, and therefore failure to fully deplete work above MMSS in the first 150 s. Given that the work above the MMSS-${{\dot{V}O}}_{2}$ power output was similar to work above laboratory-based end-test power observed elsewhere (Burnley et al. 2006; Vanhatalo et al. 2007, 2008a, 2008b, 2016), it is possible that the 3MT_R_ was paced but still a maximal effort overall (i.e., that total work done across the 180 s was maximal). Had the 3MT_R_ been performed as a sub-maximal overall effort, it is likely that work above MMSS-${{\dot{V}O}}_{2}$ would have also been noticeably low. The possibility of pacing within the 3MT_R_ is supported by the lowest 6 and 30 s power outputs being substantially lower than EP_remote_ (91 ± 5 and 97 ± 3% of EP_remote_, respectively). Periods of cycling below MMSS during the 3MT_R_ may have allowed partial recovery of the finite capacity for work above MMSS during the test (Skiba et al. 2012), and thus could have inflated the end-test power value and contributed to the overestimation of MMSS. Pacing may have been made more likely by participants being able to view elapsed time during the 3MT_R_, which is a key difference compared to laboratory 3MTs (Burnley et al. 2006; Vanhatalo et al. 2007, 2008a, 2008b).

A further key difference was the opportunity for altering the resistance to pedalling in the remote test, compared to the fixed linear factor used in previous work (Burnley et al. 2006; Vanhatalo et al. 2007, 2008a). Peak cadence achieved in the 3MT_R_ in the present investigation (110 ± 21 revs^.^min^−1^) was substantially lower than those typically achieved in the laboratory test (~ 140–155 revs^.^min^−1^), although end-test cadence was similar (Burnley et al. 2006; Vanhatalo et al. 2008b, a). Therefore, given the similarity in peak power output between our remote and previous studies of laboratory-based 3MTs, it is likely participants in the present investigation self-selected a greater pedalling resistance in the initial part of the test. Differences in the cadence profiles of the 3MT_R_ investigated here and the laboratory-based 3MT reported elsewhere may also contribute to why work above MMSS was not fully depleted in the initial 150 s of the remote test. It has previously been shown that adjusting the linear factor to produce a higher peak cadence (155 ± 12 vs. 148 ± 15 revs^.^min^−1^) resulted in a significant reduction in end-test power and total work done in a laboratory-based 3MT (Vanhatalo et al. 2008a), time-to-task failure at a constant-work rate in the severe domain was reduced when cadence was experimentally increased by 20 revs^.^min^−1^ (Nielsen et al. 2004), and mean power output during a 30-s all-out sprint was reduced by ~ 15% when performed isokinetically (100 revs^.^min^−1^) compared to isoinertially with a higher peak cadence (117 ± 14 revs^.^min^−1^) (Fuentes et al. 2013). As the rate of metabolic energy expenditure is increased at a given power output at higher pedalling cadences (Umberger et al. 2006; Brennan et al. 2019), it is possible the higher cadences achieved during laboratory trials may be necessary to fully deplete work above MMSS in the initial period of a 3MT, and therefore for end-test power to produce a valid estimate of the MMSS. This may explain why other studies have reported lower end-test power output during laboratory-based 3MTs performed at higher than preferred cadences (Wright et al. 2019), and that critical power is greater when cycling at 60 vs.100 revs^.^min^−1^ (Barker et al. 2006).

Future research may seek to determine if alteration to the 3MT_R_ instructions used in the present investigation would facilitate full depletion of work above MMSS in the initial component of the test, and therefore provide a valid estimate of MMSS. Speculatively, video conferencing in which researchers or a coach view the test and provide verbal encouragement in real time may help to reduce pacing and would also obviate the need for athletes to be able to view elapsed time. Another strategy may be to partially deplete work above MMSS prior to a 3MT_R_ with a planned severe-intensity effort. This approach is similar to what has been investigated recently in a laboratory setting, whereby a ramp test is performed prior to a 3MT (Goulding et al. 2021).

In summary, the present investigation suggests that whilst the three-minute all-out test can be performed reliably by endurance-trained cyclists in remote settings using typical indoor training set-ups, these tests overestimated the MMSS, likely due to failure to fully deplete work above end power in the initial 150 s of the test. Therefore, the 3MT_R_ protocol adopted in the present investigation should not be used for identification of the MMSS. Given that many individual athletes do not have regular access to laboratory facilities (and the on-going possibility of restricted travel to, and use of, laboratories during global pandemics), future research should explore if alterations to the 3MT_R_ protocol utilised here can produce valid estimates of the MMSS.

## Abbreviations

3MT: Three-minute all-out test
3MT_R_: Three-minute all-out test performed in a remote setting
CV: Coefficient of variation
EP_remote_: End-test power output in the remote three-minute all-out test
ES: Effect size
GET: Gas exchange threshold
MMSS: Maximum metabolic steady state
MMSS-[La^−^]: Power output at the maximum metabolic steady state defined by blood lactate concentrations
MMSS-[${{\dot{V}O}}_{2}$]: Power output at the maximum metabolic steady state defined by oxygen uptake kinetics
TWD: Total work done
${{\dot{V}O}}_{2}$: Rate of oxygen consumption
${{\dot{V}O}}_{2}$peak: Peak rate of oxygen consumption
W′: Finite work capacity above critical power
WEP_remote_: Work performed above the end-test power output in the remote three-minute all-out test
